# Exploring Target Genes Involved in the Effect of Quercetin on the Response to Oxidative Stress in *Caenorhabditis elegans*

**DOI:** 10.3390/antiox8120585

**Published:** 2019-11-25

**Authors:** Begoña Ayuda-Durán, Susana González-Manzano, Antonio Miranda-Vizuete, Eva Sánchez-Hernández, Marta R. Romero, Montserrat Dueñas, Celestino Santos-Buelga, Ana M. González-Paramás

**Affiliations:** 1 Grupo de Investigación en Polifenoles, Universidad de Salamanca, Campus Miguel de Unamuno, 37007 Salamanca, Spain; bego_ayuda@usal.es (B.A.-D.); susanagm@usal.es (S.G.-M.); evasanher@usal.es (E.S.-H.); mduenas@usal.es (M.D.); paramas@usal.es (A.M.G.-P.); 2 Instituto de Biomedicina de Sevilla, Hospital Universitario Virgen del Rocío/CSIC/Universidad de Sevilla, 41013 Sevilla, Spain; amiranda-ibis@us.es; 3 Center for the Study of Liver and Gastrointestinal Diseases (CIBERehd), Experimental Hepatology and Drug Targeting (HEVEFARM), Universidad de Salamanca, Institute for Biomedical Research of Salamanca (IBSAL), 37007 Salamanca, Spain; marta.rodriguez@usal.es

**Keywords:** quercetin, IIS pathway, *C. elegans*, oxidative stress

## Abstract

Quercetin is one the most abundant flavonoids in the human diet. Although it is well known that quercetin exhibits a range of biological activities, the mechanisms behind these activities remain unresolved. The aim of this work is to progress in the knowledge of the molecular mechanisms involved in the biological effects of quercetin using *Caenorhabditis elegans* as a model organism. With this aim, the nematode has been used to explore the ability of this flavonoid to modulate the insulin/insulin-like growth factor 1(IGF-1) signaling pathway (IIS) and the expression of some genes related to stress response. Different methodological approaches have been used, i.e., assays in knockout mutant worms, gene expression assessment by RT-qPCR, and *C. elegans* transgenic strains expressing green fluorescent protein (GFP) reporters. The results showed that the improvement of the oxidative stress resistance of *C. elegans* induced by quercetin could be explained, at least in part, by the modulation of the insulin signaling pathway, involving genes *age-1, akt-1, akt-2, daf-18, sgk-1, daf-2*, and *skn-1*. However, this effect could be independent of the transcription factors DAF-16 and HSF-1 that regulate this pathway. Moreover, quercetin was also able to increase expression of *hsp-16.2* in aged worms. This observation could be of particular interest to explain the effects of enhanced lifespan and greater resistance to stress induced by quercetin in *C. elegans*, since the expression of many heat shock proteins diminishes in aging worms.

## 1. Introduction

Quercetin (Q) is the most abundant flavonol in the human diet, being present in a wide variety of plant-derived foods, such as nuts, grapes, onions, broccoli, apples, or black tea. Dietary quercetin consumption has been associated to different health benefits, including antioxidant and anti-inflammatory effects and protection against aging-related diseases, such as cardiovascular pathologies, cancer, and neurodegenerative disorders [1,2,3]. Classically, these actions have been explained, at least in part, by its antioxidant and free-radical scavenging properties, as demonstrated in in vitro studies [4,5], although the actual mechanisms through which quercetin and other flavonoids exert their in vivo effects remain unresolved. Understanding the molecular mechanisms by which Q can exert its biological activity is important in order to develop strategies to modulate the physiological changes associated with aging that lead to chronic diseases. Although studies with quercetin and other flavonoids using in vivo models have increased in recent years, most of the knowledge on their biological activity still derives from in vitro or ex vivo findings, while assays that consider complex interactions of various processes, such as absorption, metabolism, and interaction with organs and tissues are more limited [6].

Some important molecular pathways in complex organisms can be explored using the model organism *Caenorhabditis elegans*. There is a high degree of homology between *C. elegans* and human genes involved in different processes, such as aging, apoptosis, cell signaling, cell polarity, metabolism, or cell cycle, which are conserved between mammals and the nematode [7]. In fact, *C. elegans* offers promising possibilities to study mechanisms of action and effects of secondary compounds of foods and plants, due to its simplicity of handling, conservation of metabolic pathways, and the possibility of manipulating cell signaling routes by biotechnological methods. In addition, it is not pathogenic, and no ethical boundaries exist to its experimental usage.

Different studies have evaluated the biological effects of quercetin and related compounds in *C. elegans*. In a previous work, we have shown that growing *C. elegans* in the presence of 200 µM of Q or its 3′- and 4′-*O*-methylated metabolites significantly prolonged the lifespan and increased the resistance against thermal- and juglone-induced oxidative stress [8]. Further support was obtained by checking the oxidation status of proteins in the nematode, which was greatly reduced [8]. Similar observations were also made by other authors [9,10,11,12,13]. Treatment with Q was also seen to lead to a reduction in the intracellular levels of reactive oxygen species (ROS), either in nematodes subjected to or not subjected to stress [11,13,14,15]. In general, these studies show that Q possesses a relevant biological activity in an in vivo system, which results in greater protection against oxidative processes. Regarding worm lifespan, an extension has been found in worms grown in the presence of concentrations of Q between 70 and 200 μM, while no lifespan extension was observed for Q concentrations outside that range. On the contrary, a hormetic response of the worm to this phytochemical has been demonstrated, so that the increase in the concentration of quercetin above certain levels results in decreased survival [16]. Nevertheless, the molecular mechanisms through which these effects are produced are still unclear, and different explanations, sometimes apparently contradictory, have been offered by distinct authors [9,10,11,12,13,14].

**Figure 1:**
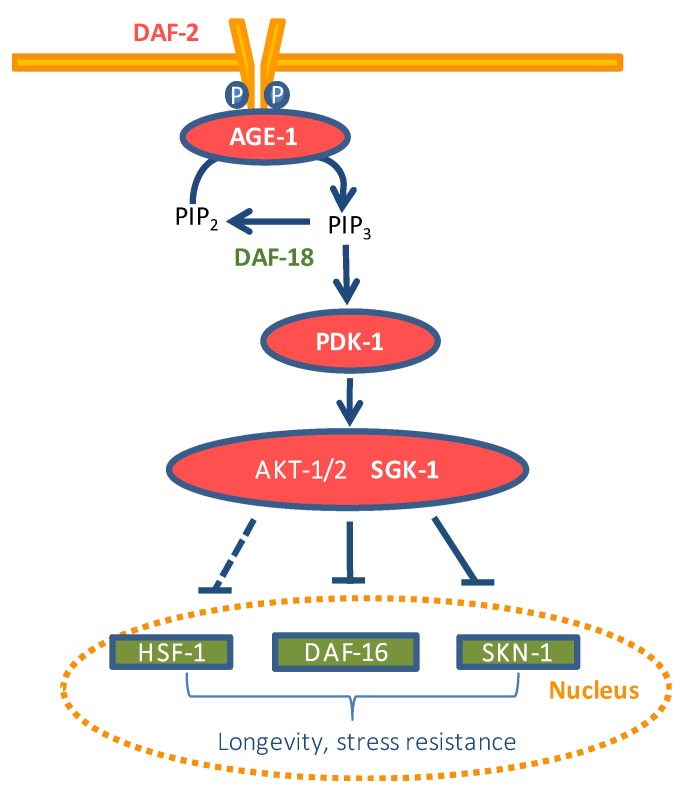
Scheme of the insulin/IGF-1 signaling pathway (IIS) in *C. elegans.* The components of the pathway that promote IIS are colored red and those that either antagonize IIS or are antagonized by IIS are colored green.

The response to oxidative stress in *C. elegans* is regulated through several pathways, including those of insulin/IGF-1 (IIS), c-Jun N-terminal kinases (JNK), and the signaling p38 MAPK (mitogen-activated protein kinases) pathway [17,18,19]. The IIS pathway (Figure 1) controls many important biological processes, including development, reproduction, metabolism, somatic maintenance, and stress resistance [20]. Insulin-like peptides binding to DAF-2, the orthologue of the insulin/IGF-1 receptor in *C. elegans* [21], activate its tyrosine kinase activity. This activation triggers a cascade of phosphorylation events through different kinases (AGE-1/PI3k, PDK-1, AKT-1/2, and SGK-1) that promote the phosphorylation-dependent cytoplasmic sequestration of the factors DAF-16/FoxO, HSF-1, and SKN-1/Nrf, preventing their transcriptional activity [22]. On the other hand, loss of insulin signaling in *C. elegans* results in several cytoprotective phenotypes resistant to both thermal and oxidative stress and also increases pathogen resistance and lifespan [23]. Flavonoids could influence cellular systems changing the expression of different genes through the modulation of distinct transcription factors, acting simultaneously on various signaling pathways, including the IIS pathway [24].

In order to contribute to elucidate the mechanisms involved in the effects of flavonoids, in this work, the ability of quercetin to modulate the insulin/IGF-1 signaling pathway (IIS) has been explored. Specifically, the influence on the resistance to thermally-induced oxidative stress has been assessed using *C. elegans* strains with loss-of-function mutations in genes of the IIS pathway (i.e., *daf-2, age-1, daf-16, akt-1, akt-2; sgk-1, hsf-1, skn-1*, and *daf-18*). Additionally, the ability of Q to modify the expression of some genes related to stress, namely *daf-16, hsf-1, skn-1, hsp-16.2, hsp-70, sod-3*, and *gst-4*, has also been determined by RT-qPCR or using the GFP fluorescent reporter in *C. elegans* transgenic strains.

## 2. Materials and Methods

### 2.1. Standards and Reagents 

Quercetin (Q), ampicillin sodium salt, nistatine, agar, yeast extract, fluorodeoxyuridine (FUdR), phosphate-buffered saline (PBS), cholesterol, and 2-mercaptoethanol, were purchased from Sigma-Aldrich (Madrid, Spain). Dimethyl sulfoxide (DMSO) was obtained from Panreac (Barcelona, Spain). SYBR^®^ SelectMaster Mix and high-capacity cDNA reverse transcription kit were from Applied Biosystems (Carlsbad, CA, USA), and the Illustra™ RNAspin mini isolation kit was from GE Healthcare (Buckinghamshare, UK).

### 2.2. Strains and Maintenance Conditions

The wild-type strain N2 and the mutant strains VC475, *hsp-16.2(gk249)* V; CB1270, *daf-2 (e1370)* III; TJ1052, *age-1(hx546)* II; CF1038, *daf-16(mu86)* I; CB1375, *daf-18(e1375)* IV; BQ1, *akt-1(mg306)* V; KQ1323, *akt-2(tm812) sgk-1(ft15)* X; PS3551, *hsf-1(sy441)* I; EU1, *skn-1(zu67)* IV*/nT1(unc-?(n754)let-?)* (IV;V); CF1553, *muls84 ((Psod-3::gfp))*; TJ356, *zIs356 (Pdaf-16::daf-16::gfp; rol-6 (su1006))* IV; CL2166, *dvIs19 ((Pgst-4::gfp::NLS; rol-6 (su1006))* III; AM446, *rmIs223 (Phsp70::gfp; rol-6(su1006))*; CL2070, *dvIs70 (Phsp-16.2::gfp; rol-6 (su1006))*, as well as the *E. coli* OP50 bacterial strain, were obtained from the *Caenorhabditis* Genetics Center (CGC) at the University of Minnesota (Minneapolis, MN, USA). Worms were routinely propagated at 20 °C on nematode growth medium (NGM) plates with *E. coli* OP50 as a food source.

Synchronization of worm cultures was achieved by treating gravid hermaphrodites with bleach: 5N NaOH (2:1). Eggs are resistant whereas worms are dissolved in the bleach solution. The suspension was shaken with a vortex mixer during 1 min and kept for a further minute on rest, this process was repeated five times. The suspension was centrifuged (2 min, 9500 *g*). The pellet containing the eggs was washed six times with an equal volume of buffer M9 (3 g KH_2_PO_4_, 6 g Na_2_HPO_4_, 5 g NaCl, 1 mL 1M MgSO_4_, H_2_O to 1 L). Quercetin solution (200 mM) in DMSO was added to the nematode growth medium during its preparation to get a 200 μM final concentration on the plates. Control plates were also prepared without the flavonoid but containing the same volume of DMSO (0.1% DMSO, *v/v*). Around 100 to 300 μL of the M9 with eggs (depending on eggs Øconcentration) were transferred and incubated on NGM agar plates with or without Q. When the worms reached the L4 stage, they were transferred to new plates with or without Q but also containing FUdR at a concentration of 150 μM to prevent reproduction and progeny overgrowth. The worms were transferred every 2 days to fresh plates with FUdR for the different treatments (with or without Q) until they reached the day of the assay.

### 2.3. Stress Assays

Oxidative stress in worms was induced by subjecting the animals to 35 °C heat-shock treatment. Worms were incubated at 20 °C on NGM-*E. coli* OP50 plates with or without Q until days 2 and 9 of adulthood. Then they were transferred with a platinum wire to agar plates Ø 35 mm, 20 worms per plate) and switched to 35 °C for 4, 6, or 8 h. The time was decided depending on the thermotolerance of the specific worm strain used in the assay, which was previously checked. After that time, dead and alive nematodes were counted. In the studies involving the use of worm mutants, in addition to the mutant control, a parallel control using N2 wild-type (WT) worms was also included. For the assays, ten plates were used per treatment containing 20 worms per plate, resulting in a total of 200 worms, although a small percentage of worms was usually lost in the score. Only in the case of the *skn-1* mutant (EU1) were 300 worms (20 worms per plate/15 plates) used. According to the information supplied by the CGC, the work with this mutant required special considerations, as only the homozygote worms with WT appearance are considered for the assay, while the uncoordinated heterozygote worms are only employed to maintain the lineage. To have reproducible results in the heat shock assays, that is, to have the smallest possible temperature oscillations, some precautions were followed, in agreement with the guidelines stated in the reference paper [25]. During assays, the door of the incubator was only opened when the survival rate had to be measured. The plates were not stacked inside the incubator, they were placed in a row on the same shelf of the incubator, leaving enough space between them for the air to circulate properly. The temperature was controlled with the incubator’s own thermostat and with an external thermostat with sensors inside the incubator to monitor possible temperature oscillations.

### 2.4. RT-qPCR Assays

Adult worms of the N2 *C. elegans* strain were treated with or without 200 μM of Q for 4 days. The worms were collected with M9 buffer, centrifuged at 10,000 *g* for 1 min, and the pellet was dissolved in 300 µL of M9, to which 3.5 µL of 2-mercaptoethanol was added. Total RNA was extracted using the RNAspin Mini RNA Isolation Kit (GE Healthcare). In order to maximize cell breakage, in the first stage of the extraction, 10 stainless-steel beads (2 mm) were added. The mixture was vortex shaken vigorously and further homogenized in a Thermo Savant FastPrep 120 Cell Disrupter System, with a speed of 5.5 m/s and run time duration of 10 s, five times. cDNA was produced with high-capacity cDNA reverse transcription kit (Applied Biosystems) using 2 µg of total RNA per reaction. The expression of mRNA was assessed by quantitative real-time PCR, using SYBR green as the detection method. The gene expression data were analyzed using the comparative 2^−ΔΔCt^ method, with *act-1* as the normalizer [26]. Nine independent experiments were performed. *Act-1* was used as a normalizer both in the assays carried out in non-stressed and stressed worms. The information related to gene-specific primers used in this work can be found in the Supplementary Table S1.

### 2.5. Fluorescence Quantification and Visualization

Synchronized L1 larvae expressing an inducible green fluorescent protein (GFP) reporter for *gst-4, hsp-16.2, hsp-70, sod-3*, and *daf-16* genes were grown on NMG plates in the presence or absence of Q until the day of the assay, when they were subjected to or not subjected to thermally-induced oxidative stress (35 °C, 1 h). The precise day of assay was defined when a higher intensity of the fluorescence was observed after carrying out a screening with the different strains throughout the life of the worm, namely, day 3 in *gst-4* and day 5 in *hsp-70*. For the remaining strains, as no clear increase in the fluorescence was observed, the assessment was made in young (day 2 of adulthood) and older adult worms (day 9 of adulthood). In the cases of *hsp-16.2* and *hsp-70* reporter strains, after thermal stress, the worms were allowed to recover at 20 °C for 2 or 3 h respectively, before pictures were taken. The expression of *gst-4, hsp-16.2, hsp-70*, and *sod-3* was measured by quantifying the fluorescence of the GFP reporter. To analyze the subcellular localization of the DAF-16::GFP reporter, worms were classified as diffuse cytoplasmic, intermediate cytoplasmic/nuclear, and strong nuclear translocation. Approximately 35 randomly selected worms for each experiment were mounted in a 5 µL drop of 10 mM levamisole (except for DAF-16::GFP in 2% sodium azide) on a 3% agarose pad covered with a coverslip. All fluorescence determinations were done in an Olympus BX61 fluorescence microscope equipped with a filter set (excitation 470 ± 20 mn, emission 500 ± 20 nm) and a DP72 digital camera coupled to CellSens Software for image acquisition and analysis. ImageJ software was used to quantify fluorescence intensity. Three independent experiments were performed per assay and reporter strain.

### 2.6. Statistical Analysis

The statistical analyses were performed using the PC software package SPSS (version 23.0; SPSS Inc., Chicago, IL, USA). Analysis of variance (ANOVA) was applied for multiple comparisons of values to determine possible significant differences between treated and control groups. To analyze survival to thermal stress, contingency tables were prepared, and statistical significance was calculated using the Chi Square Test.

## 3. Results and Discussion

### 3.1. Assays in Wild Type and Mutant Worms

**Figure 2:**
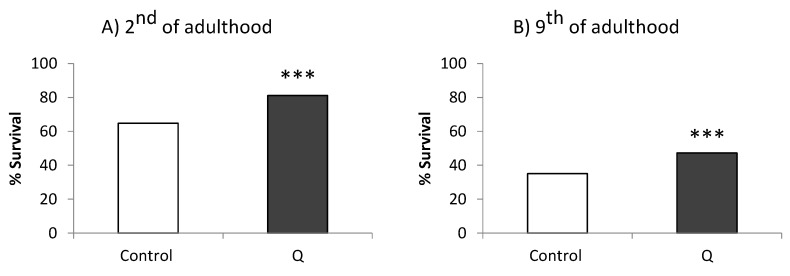
Percentages of survival following thermal stress (35 °C, 8 h) applied at days (**A**) 2 and (**B**) 9 of adulthood in N2 wild-type *C. elegans* strain not treated (controls) and treated with Q (200 µM). Statistical significance was calculated using the Chi Square Test (200–300 individuals per assay in both controls and treated worms). The asterisks (***) indicate significant differences at *p* < 0.001.

An enhancement in the survival was observed in N2 wild-type worms treated with Q 200 µM after being subjected to thermal stress compared with non-treated controls. Specifically, in the assays carried out on the second day of adulthood, the average proportion of living worms after stress was 64.78% in the control group and 81.11% in Q-treated worms (*p* = 0.000), while on the ninth day of adulthood, the survival rate was 35.1% in untreated worms and 47.2% in treated animals (*p* = 0.001) (Figure 2).

**Figure 3:**
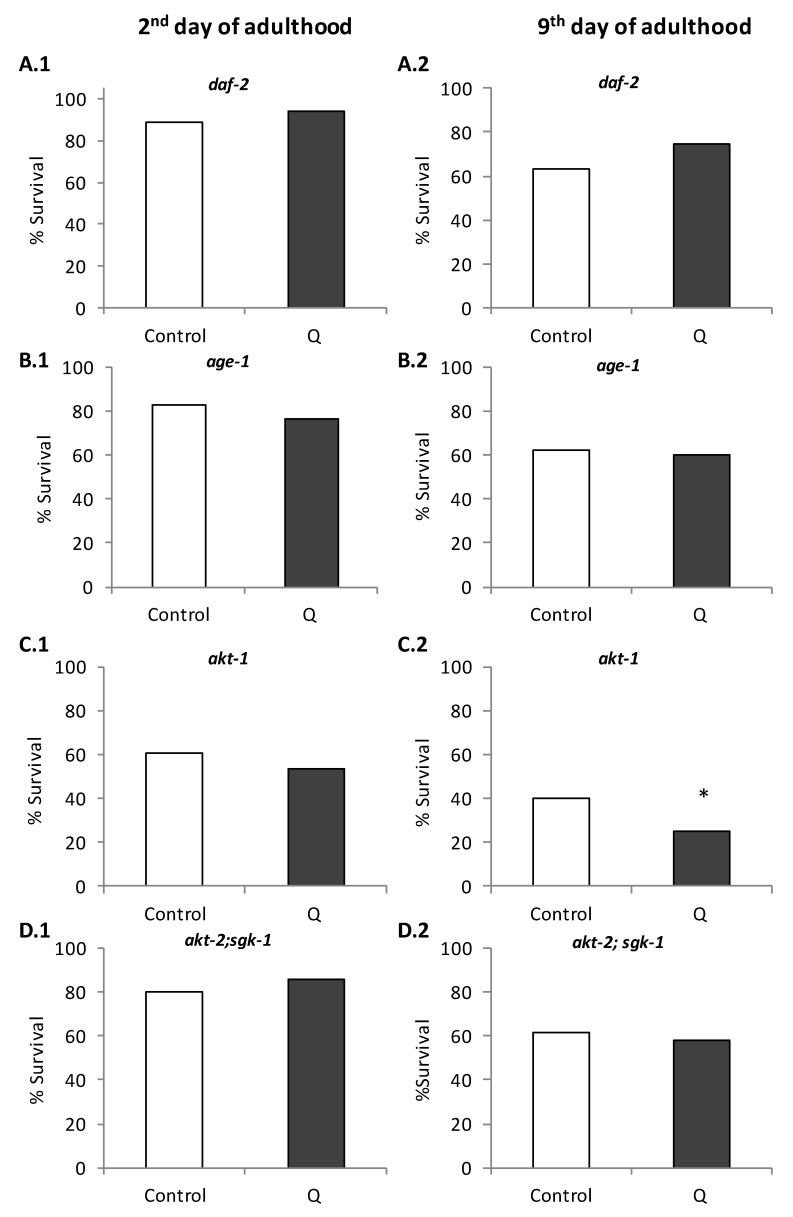
Percentages of survival following thermal stress (35 °C, 8 h) applied at days 2 (**A–D.1**) and 9 (**A–D.2**) of adulthood in different long-lived *C. elegans* mutants from the IIS pathway: *daf-2(e1370)* (**A**), *age-1(hx546)* (**B**), *akt-1(mg306)* and *akt-2(tm812)* (**C**); *sgk-1(ft15)* (**D**) cultivated in the absence (controls) and presence of Q (200 μM). Statistical significance was calculated using the Chi Square Test. In both controls and treated worms, ten plates were used per assay containing 20 worms per plate (i.e., 200 worms in total). The asterisk (*) indicates significant differences at *p* < 0.05.

The molecular mechanisms involved in this enhancement of thermotolerance were explored, checking the ability of Q to modulate the stress resistance in *C. elegans* mutant strains. Initially, the effect of Q on thermal stress resistance was evaluated in worms carrying loss of function mutations in genes of the IIS pathway, namely *age-1(hx546)*, *akt-1(mg306)*, *daf-2(e1370),* and the double mutant *akt-2(tm812); sgk-1(ft15)*, all of them long-lived and with greater resistance to stress than the wild-type strain. Similar to the wild-type, mutant worms were subjected to a thermal shock (35 °C, 6–8 h) on days 2 and 9 of adulthood. The obtained results showed that the stress resistance was not increased in any of the mutant worms treated with quercetin at either day 2 or 9 (Figure 3). These results suggested that *age-1, akt-1, akt-2, sgk-1*, and *daf-2* were necessary to mediate stress resistance induced by Q in *C. elegans*.

Pietsch et al. [11] also studied the effect of Q in some of these mutants. As in our case, they did not find an improvement in the survival after thermal stress in *daf-2* and *age-1* mutants treated with 200 μM quercetin, concluding that that those genes were required to mediate the stress-protective effects of the flavonol. However, they found that the treatment with Q unexpectedly produced an improvement in the resistance to stress in the *akt-2* mutant, a gene located downstream of DAF-2 and AGE-1 in the IIS pathway. They proposed that this could be explained because SGK-1 was more important for resistance to stress than the AKT kinases [11]. In the present study, no increase in the survival after thermal stress was observed in the double-mutant *akt-2;sgk-1* treated with Q (Figure 3), suggesting that *sgk-1* could actually be necessary to improve the resistance to stress by quercetin. Similarly, no changes in the survival after stress were produced in Q-treated *akt-1* mutants, a gene that encodes an ortholog of serine/threonine kinase AKT/PKB and interacts with the IIS pathway. This indicates that the kinase *akt-1* was also necessary for the improvement in the resistance to stress in *C. elegans.*

**Figure 4:**
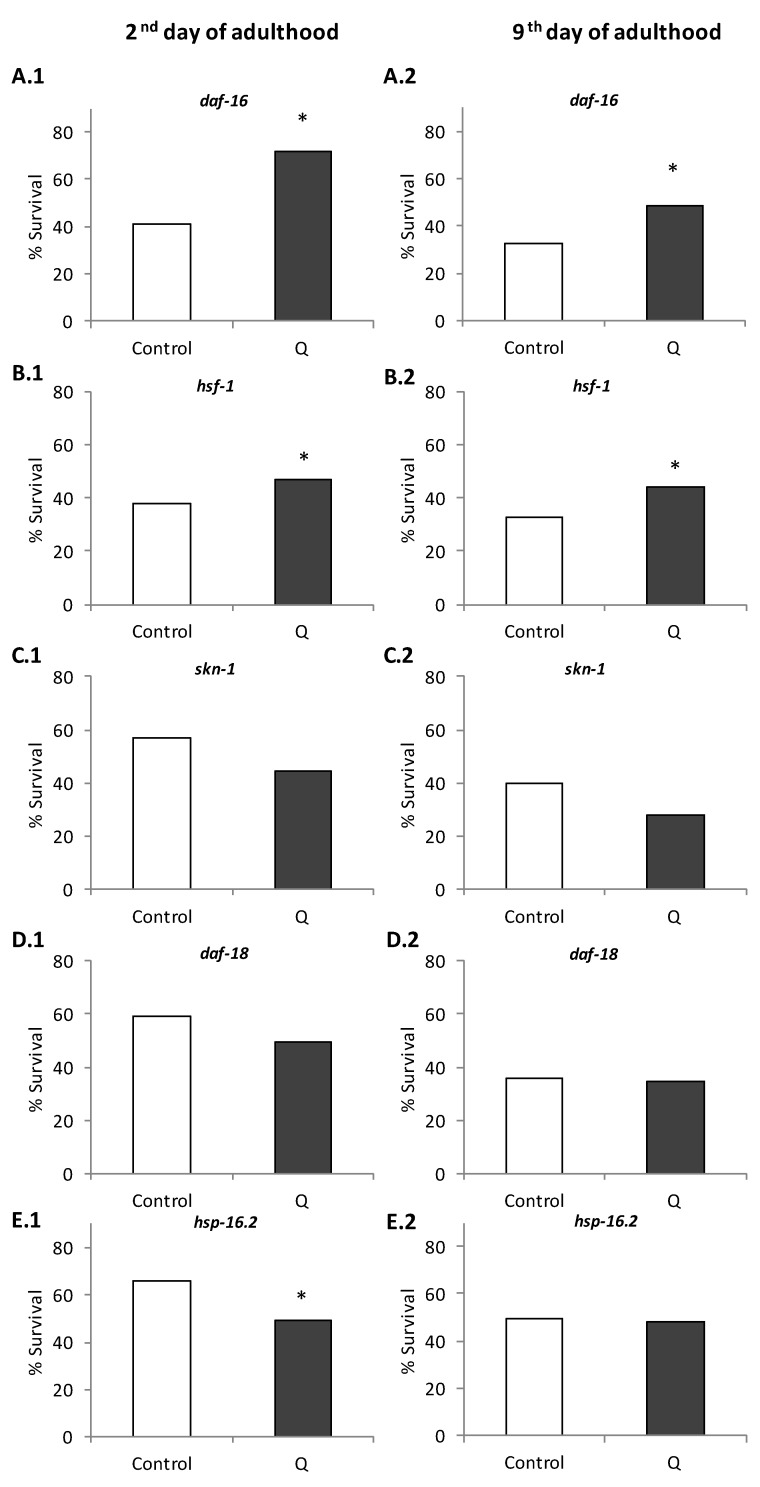
Percentages of survival following thermal stress (35 °C, 6 h; except for *hsp-16.2* (35 °C, 4 h)) applied at days 2 (**A–E.1**) and 9 (**A–E.2**) of adulthood in *daf-16 (mu86)* (**A**), *hsf-1(sy441)* (**B**), *skn-1(zu67)* (**C**), *daf-18(e1375)* (**D**) *and hsp-16.2(gk249)* (**E**) mutants cultivated in the absence (controls) and presence of Q (200 μM). Statistical significance was calculated using the Chi Square Test. In both controls and treated worms, ten plates were used per assay containing 20 worms per plate (i.e., 200 worms in total), but in the case of the *skn-1* mutant, 300 worms (20 worms per plate/15 plates) were used. The asterisk (*) indicates significant differences at *p* < 0.05 on comparing with control.

The resistance to thermal stress in response to Q was also studied in *daf-16, hsf-1, skn-1, daf-18*, and *hsp-16.2* mutant worms. The transcription factors DAF-16, HSF-1, and SKN-1 produce changes in the expression of several genes in response to a reduced IIS pathway. The genes regulated by these transcription factors are functionally relevant, including stress response genes, such as catalases, glutathione-*S*-transferases, metallothioneins, and genes that encode antimicrobial peptides, chaperones like *hsp-16.2*, apolipoproteins, and lipases [27]. As it can observed in Figure 4, the treatment with Q did not improve the survival in *daf-18, skn-1*, and *hsp-16.2* mutants exposed to thermal stress, suggesting that these genes would be necessary to explain the effect of Q in worm resistance against stress. However, the treatment with Q continued to produce an improvement in the resistance to thermal stress in *daf-16* and *hsf-1* mutants, indicating that the effect of Q in *C. elegans* was independent of these genes. Similar results on the influence of Q were obtained in young and older adults in the studied mutants (Figure 4).

### 3.2. q-RT-PCR Analyses

**Figure 5:**
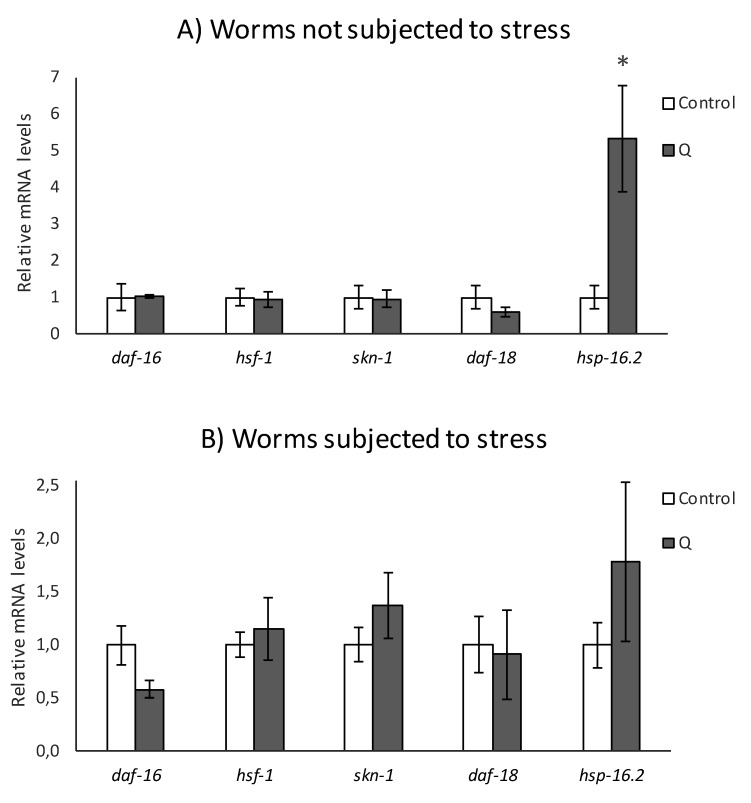
Effect of Q on the expression of *daf-16*, *hsf-1*, *skn-1, daf-18*, and *hsp-16.2* genes in N2 *C. elegans* cultivated in the absence (controls) and presence of Q (200 μM) (**A**) under non-stressed conditions or (**B**) after subjecting them to thermal stress. The expression level was determined by RT-qPCR. *act-1* was used as housekeeping control. Nine independent experiments were performed. The results are presented as the mean values ± Standard Error of the Mean (SEM). Statistical significance was calculated using by one-way analysis of variance (ANOVA). The asterisk (*) indicates significant differences at *p* < 0.05.

The influence of quercetin on some transcription factors and target genes of the IIS pathway was also explored by quantification of the expression of *daf-16, hsf-1, skn-1, daf-18*, and *hsf-16.2* by RT-qPCR in wild type worms, both submitted and not submitted to thermal stress (5 h, 35 °C) after growing 4 days in the presence of the flavonol. As it can be seen in Figure 5, the treatment with Q did not modify the expression of *daf-18* either after subjecting or not subjecting the worms to thermal stress. However, the results previously obtained with the mutants (Figure 4) indicated that *daf-18* could be involved in the protective effects of quercetin against stress. A possible explanation to this apparent contradiction could be that the result obtained in the *daf-18* mutant is rather reflecting the involvement of the protein kinase AGE-1, as the DAF-18/phosphatase and tensin homolog (PTEN)protein is responsible for dephosphorylating and inhibiting AGE-1/PI3K, counteracting its activity [21]. Another possibility is that DAF-18 was regulated by quercetin at the post-transcriptional or activity level.

Similarly, the expression of *hsf-1* was not modified by the treatment with Q, neither in normal growing conditions nor after application of thermal stress (35 °C, 5 h) (Figure 5). This result would confirm that the heat transcription factor *hsf-1* is not involved in the effects of Q on stress resistance, supporting the above-described observations on *hsf-1* mutants (Figure 4). In a previous study on *hsf-1* loss of function mutants, Fitzenberger et al. [28] also found that this gene was not required to explain the ability of Q to prevent the glucose-induced reduction of survival in *C. elegans.*

The treatment with Q did not produce changes in the expression of *daf-16* (Figure 5). Together with the findings obtained in the assays with mutants, these results seem to confirm that the effect of Q on worm stress resistance is independent of *daf-16*. Other authors had already studied the involvement of *daf-16* in the effects of Q using knockout worms [10,11,13], finding that Q continued improving longevity and resistance to thermal and hydrogen peroxide-induced oxidative stress in *daf-16* mutants, which suggest that this gene was not essential for the effects of quercetin, observations that are consistent with the results obtained herein, either in worms subjected or not subjected to stress.

SKN-1 is the homologue of Nrf-2 transcription factor, which regulates oxidative stress response and lifespan, mobilizing the conserved phase 2 detoxification response [29]. No differences were found in the expression of *skn-1* between worms treated or not treated with Q and not subjected to stress (Figure 5A), which seems in agreement with the observations of Pietsch et al. [11], who reported that quercetin induced an increase in the lifespan of *skn-1* mutants under normal growth conditions, indicating that the effects of this flavonoid in the absence of stress are independent of that gene. However, the results obtained with the *skn-1* mutants (Figure 4) indicated that this gene is a mediator in the protective effects of Q against stress. Tullet et al. [29,30] suggested that SKN-1 is a transcriptional co-regulator of DAF-16 regarding resistance to oxidative stress and the expression of detoxification genes in response to a reduced IIS signal, but it extends worm half-life independently of DAF-16. This dual function of *skn-1* might be coherent with the results observed herein, where Q did not change the *skn-1* expression in absence of stress, although this gene seems to be necessary in the improvement of resistance to thermal stress mediated by Q.

The influence of Q on thermal shock proteins HSP-16.2, whose expression is influenced by the IIS, was also studied. As shown in Figure 5, the treatment with Q induced an increase in the expression of *hsp-16.2*, either without or with stress. However, the differences were more noticeable in the absence of stress, which could possibly be explained by the already strong induction of HSP-16.2 caused by the thermal stress, which would make the effect induced by Q treatment less relevant. Together with the results in the *hsp-16.2* mutants, where a decrease in the resistance to thermal stress was observed in quercetin-treated worms (Figure 4), it appears that thermal shock proteins can be involved in the protective effects of the flavonol against stress.

All in all, the obtained results showed that, at least in part, the IIS pathway would be involved in the improvement to thermotolerance induced by Q, entailing the genes *age-1, akt-1, akt-2, sgk-1, daf-2, daf-18, skn-1*, and *hsp-16.2*, but independent of *daf-16* and *hsf-1*. In addition, the involvement of these genes was not modified by the age of the worm. Previous studies by our group with epicatechin also showed that the enhanced stress resistance induced by this flavan-3-ol in *C. elegans* was also mediated by the IIS pathway, although it did not necessarily involve the same genes, as in that case the expression levels of the main transcription factors of the pathway (*daf-16, skn-1*, and *hsf-1*) were modified by the compound [31]. Actually, even compounds belonging to the same flavonoid class seem to act through different mechanisms. Thus, the lifespan extension produced by quercetin-3-*O*-diglucoside in *C. elegans* was explained by upregulation of the genes *daf-2, old-1, osr-1*, and *sek-1*, whereas no modification was produced in the expression of *daf-16, age-1*, and *sir-2.1* [12]. However, assays with a flavonol-rich extract obtained from *Baccharis trimera* concluded that the improvement in the stress resistance was independent of several stress-related signaling pathways (p38, JNK, and ERK) and transcription factors SKN-1 and DAF-16 [32].

### 3.3. Assays with Fluorescent Reporters

The insulin signaling pathway transmits signals in response to the environmental conditions that could change the expression of different genes related to stress and longevity, thus regulating important processes such as aging, metabolism, or dauer formation. The expression of some such genes, namely those encoding heat shock proteins (*hsp*-16.2 and *hsp*-70) and antioxidant enzymes (*sod*-3 and *gst-4*), was assessed in order to gain further insight into the mechanisms of action underlying the effects of Q on the modulation of lifespan and stress in *C. elegans*. With this aim, transgenic strains that express GFP under the control of *gst-4, sod-3, hsp-16.2*, and *hsp-70* promoters were employed. GFP expression levels were analyzed in animals grown in the presence or absence of quercetin under non-stress conditions for *Pgst-4::gfp* and *Psod-3::gfp* reporters, whereas for *Phsp-70::gfp* and *Phsp-16.2::gfp* reporters, worms were previously submitted to a heat shock (35 °C, 1 h) and further allowed to recover for 3 h (*hsp-70*) or 2 h *(hsp-16.2*) at 20 °C.

**Figure 6:**
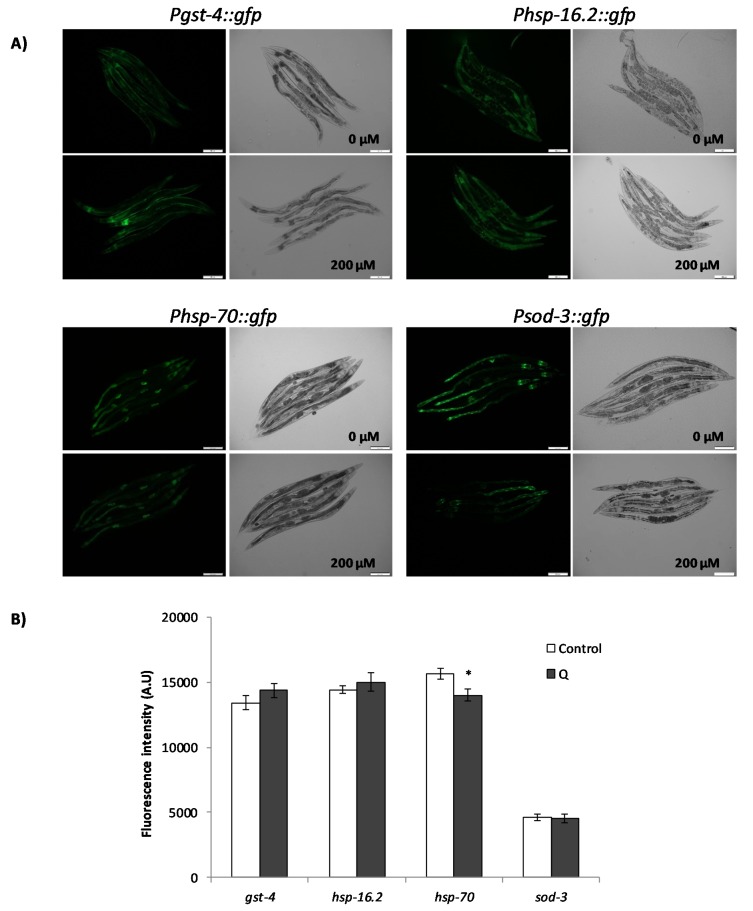
Effect of Q on the expression of *gst-4, hsp-16.2, hsp-70*, and *sod-3* after cultivation of *C. elegans* in the absence and presence of Q (200 μM). (**A**) Representative fluorescence images of control and Q-treated worm strains. (**B**) Quantification of the relative fluorescence intensities of transgenic worms. Total green fluorescent protein (GFP) fluorescence of each whole worm was quantified using Image J software. Three independent experiments were performed. The results are presented as the mean values ± SEM. Approximately 35 randomly selected worms from each set of experiments were examined. Differences compared with the control (0 μM Q, 0.1% dimethyl sulfoxide (DMSO)) were considered statistically significant at * (*p* < 0.05) by one-way ANOVA.

**Figure 7:**
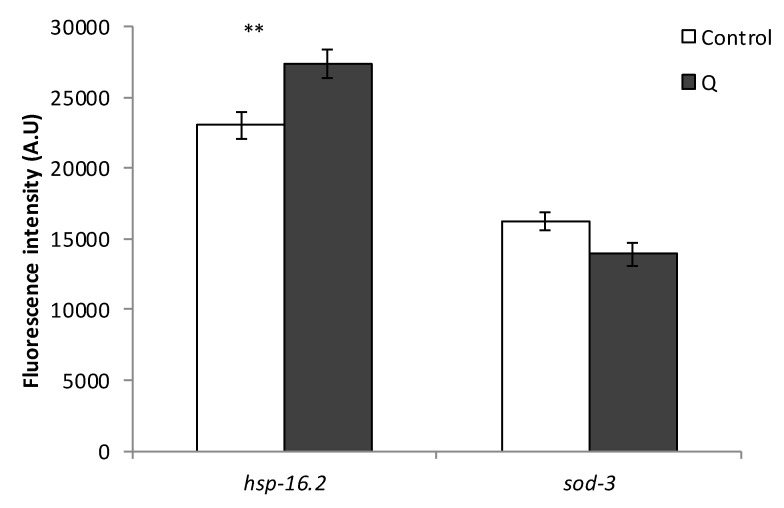
Effect of Q on the expression of *hsp-16.2* and *sod-3* in old worms (day 9 of adulthood) after cultivation of *C. elegans* in the absence and presence of Q (200 μM). Total GFP fluorescence of each whole worm was quantified using Image J software. Three independent experiments were performed. The results are presented as the mean values ± SEM. Approximately 35 randomly selected worms from each set of experiments were examined. Differences compared with the control (0 μM Q, 0.1% DMSO) were considered statistically significant at ** (*p* < 0.01).

As it can be seen in Figure 6, the treatment with Q did not produce an increase in the expression of any of the studied genes (*gst-4, sod-3, hsp-16.2*, and *hsp-70*) in young worms of reproductive age. Indeed, there was even a decrease in the expression of *hsp-70* in the worms treated with Q (*p* = 0.018). The expression of *hsp-16.2* and *sod-3* was also studied using reporter strains in older worms (day 9), in order to establish if the mechanism of action underlying the effects of Q was dependent on the age of the worm. Similar results were obtained regarding the expression of *sod-3* (Figure 7) as compared to younger worms (Figure 6), finding no differences in the expression by the treatment with Q. However, the expression of *hsp-16.2* was increased by the treatment with Q at day 9 (*p* = 0.008) (Figure 7), which was not observed at day 2 (Figure 6).

Some of these genes have already been studied by other authors using fluorescent reporters to explore worm response to Q, specifically *gst-4* and *sod-3*. Opposed results were reported regarding the effects of Q on *sod-3*. Whereas, Kampkötter et al. [9] observed a decrease in the expression of *sod-3* in worms exposed to 100 μM Q, Grünz et al. [13] found that growing the worms in the presence of 100 μM of Q produced a significant increase in *sod-3* expression. As for the present study, no significant changes were detected in the expression of *sod-3* after treatment with 200 μM of Q in any of the two days studied. On the other hand, Kampkötter et al. [14] observed that the expression of *gst-4* was not modified by Q 100 μM under normal growth conditions, although it decreased the expression of *gst-4* when worms were subjected to oxidative stress with juglone 20 μM [14]. Those results would be in agreement with the observations made herein, where no increase in the expression of *gst-4* was found under normal growth conditions (200 μM Q).

The results for *Phsp-16::gfp* obtained at different ages of the worms could help to understand the effects of increased lifespan and improvement of resistance to stress induced by Q in *C. elegans*, since many heat shock proteins are regulated positively at the beginning of adult life to further decrease throughout life [33]. The obtained result might indicate that this decrease could be reversed by the treatment with Q. It is pertinent to indicate that in previous studies on longevity, greater survival started to be observed from approximately day 8 onwards in worms treated with quercetin in relation to non-treated worms [8], suggesting changes in *C. elegans* metabolism favored by prolonged exposure to the flavonol, among which, the upregulation of some heat shock proteins could be involved. Actually, the increased lifespan and thermotolerance observed in certain mutants, such as the long-lived *age-1*, have been explained by an increase in the regulation and accumulation of HSP-16 [34]. The involvement of *hsp-16.2* was also supported by the increase in the expression of *hsp-16.2* found in the RT-qPCR studies (Figure 5) and the loss of the improvement in resistance to thermal stress in the *hsp-16.2* mutant (Figure 4). On the other hand, the results showed a decrease in the reporter of *hsp-70* in worms treated with Q (Figure 6). Differences in the expression of genes encoding distinct heat shock proteins, were also found by Pietsch et al. [15], observing that Q produced an increase in the expression of *hsp-3, hsp-12.6, hsp-16.1*, and *hsp-16.41*, but a decrease of *hsp-70* and *hsp-17*.

**Figure 8:**
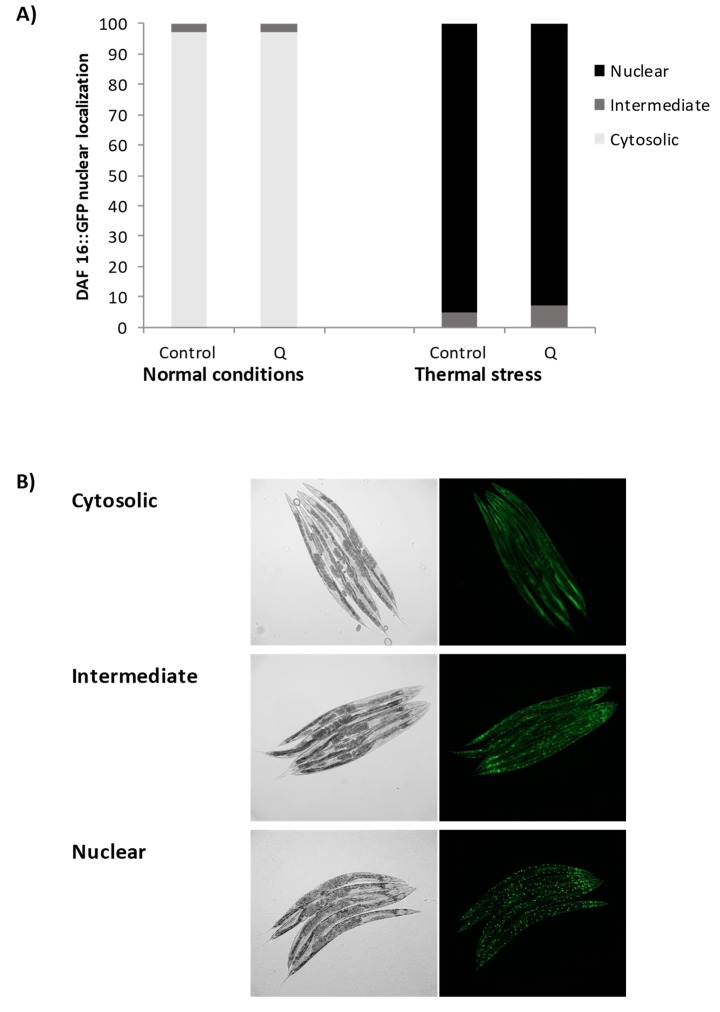
(**A**) Effect of Q on DAF-16::GFP subcellular distribution and (**B**) representative pictures of the subcellular location of DAF-16::GFP, i.e., cytosolic, intermediate and nuclear. Transgenic worms expressing the fusion protein DAF-16::GFP were cultivated in the absence (controls) and presence of Q (200 μM) after subjecting or not subjecting the worms to thermal stress and evaluated at day 2 of adulthood.

The modulation of the subcellular localization of the DAF-16 forkhead transcription factor was examined using a transgenic strain expressing a fusion protein DAF-16::GFP. The treatment with Q did not affect the translocation of DAF-16 to the nucleus with respect to the control worms, neither under normal growth conditions nor after thermal stress (Figure 8). The localization of DAF-16 was also studied in older worms (day 9), obtaining similar results that at day 2 regarding its subcellular localization.

As discussed above, the treatment with Q did not produce an increase in the expression of *daf-16* in any of the assayed conditions, while it led to an increase in the resistance to thermal stress on *daf-16* mutant strains. All these results indicate that the effect of this flavonol in the improvement of worm resistance to stress is independent of *daf-16*. A similar conclusion has been obtained by some authors [10,11], although others observed greater translocation of DAF-16 from the cytosol to the nucleus following Q treatment [9,13,14]. In this respect, Saul et al. [10] suggested that the translocation of DAF-16 to the nucleus in response to quercetin could be more of a circumstantial effect than proof of an underlying longevity mechanism. In fact, although DAF-16 is a key factor in the control of stress response and longevity [17,35,36], it has also been pointed out that its translocation to the nucleus does not guarantee a longer lifespan, suggesting that DAF-16 would not necessarily act only in the regulation of longevity [37,38].

The finding that the effects of Q can be independent of *daf-16* but dependent on *daf-2* and other components of the IIS, such as *akt-1*, *age-1*, and *akt-2*/*sgk-1*, could appear surprising. Nevertheless, Hekimi et al. [37] and Lin et al. [39] pointed out that when the IIS pathway is altered in response to different environmental signals, besides the stress resistance genes, other unidentified signals are regulated by *daf-2*, which are not dependent on *daf-16*, but that are also essential for the extended longevity in *daf-2* mutants.

In the end, the obtained results indicated that two key transcription factors that could be involved in the expression of heat shock proteins, DAF-16 and HSF-1 [40], were not related with the effects exerted by Q on *C. elegans* (Figure 4 and Figure 5). Actually, it is described that HSF-1 is not a key factor for all HSPs [41]. Mertenskötter et al. [42] showed that in the expression of chaperone genes, protein biosynthesis and protein degradation was positively influenced by the MAPK pathway and established the importance of this pathway in heat stress responses, possibly by a PMK-1-mediated activation of the transcription factor SKN-1 in *C. elegans.* Other authors have also found that components of related pathways, such as UNC-43, SEK-1, and OSR-1 are involved in the molecular mechanisms of the response to quercetin and other polyphenols [11,43,44]. Thus, the MAPK pathways could also be implicated in the effects of quercetin, which might contribute to explain the role of SKN-1 and the activation of certain HSPs observed in the present study.

## 4. Conclusions

The enhancement of the oxidative stress resistance of *C. elegans* induced by Q could be explained, at least in part, by the modulation of the insulin signaling pathway, namely the genes *age-1, akt-1, akt-2, daf-18, sgk-1, daf-2, skn-1*, and *hsp-16.2*. However, this effect would be independent of *daf-16* and *hsf-1*. The implication of *daf-2* and not of *daf-16* in the observed effects seems to reinforce the idea that there are signals regulated by *daf-2* independent of *daf-16* that are also essential for the extension of longevity and resistance to stress.

The expression of *skn-1* was not changed by the treatment with Q when worms were submitted to stress, but the results obtained in the *skn-1* mutants pointed out that it was necessary to mediate the resistance to thermal stress. It has been reported that SKN-1 promotes longevity by a different mechanism to the protection against oxidative damage [29,30], and also that the increase of lifespan by Q was maintained in *skn-1* mutants [11]. This could explain why quercetin does not alter *skn-1* expression under normal conditions, while this gene seems involved in the increased resistance to stress induced by Q.

The studies with transgenic strains showed that Q did not produce an increase in the expression of the antioxidant enzymes GST-4 and SOD-3, nor of the heat shock proteins HSP-16.2 and HSP-70 in young worms, whereas an increase was produced in the expression of HSP-16.2 in aged worms. This observation could be important to explain, at least in part, the effects of enhanced lifespan and greater resistance to stress produced by Q in *C. elegans*, since the expression of many heat shock proteins diminishes throughout worm life, a process that might be counteracted by this flavonol.

In summary, the network of signaling pathways that could be modulated by quercetin and other flavonoids is diverse and complex, and the molecular mechanisms of action can vary depending on the compound. For instance, while DAF-16 and HSF-1 transcription factors were previously demonstrated to be involved in the effects of epicatechin [31], they do not seem to be required in the stress resistance effects of Q. In this work, just the insulin signaling pathway has been explored, but other routes are surely involved, which should be considered in future studies.

## Supplementary Materials

The following are available online at https://www.mdpi.com/2076-3921/8/12/585/s1, Table S1: Oligonucleotide sequence of primers used to determine the expression levels of *C. elegans* genes by RT-QPCR.
