# Supplementary material for: Exploring Target Genes Involved in the Effect of Quercetin on the Response to Oxidative Stress in Caenorhabditis elegans
Source: Antioxidants (Basel). 2019 Nov 25;8(12):585. doi: 10.3390/antiox8120585 (PMC6943653; doi:10.3390/antiox8120585)
Supplement: Supplementary file 1 [file antioxidants-08-00585-s001.pdf]

Table S1: Oligonucleotide sequence of primers used to determine the expression levels of *C. elegans* genes by RT-QPCR

Protein	Gene	Forward (5′-3′)	Reverse (5′-3′)	Size bp	Accession number	References
ACT-1	*act-1*	CCAGGAATTGCTGATCGTATG	GGAGAGGGAAGCGAGGATAG	131	NM_073418.6	[1]
SKN-1	*skn-1*	AGTGTCGGCGTTCCAGATTTC	GTCGACGAATCTTGCGAATCA	109	NM_171345.5	[2]
DAF-16	*daf-16*	CCAGACGGAAGGCTTAAACT	ATTCGCATGAAACGAGAATG	148	NM_001026422.5	[3]
HSF-1	*hsf-1*	GAAATGTTTTGCCGCATTTT	CCTTGGGACAGTGGAGTCAT	99	NM_060630.4	[4]
HSP-16.2	*hsp-16.2*	CTGCAGAATCTCTCCATCTGAGTC	AGATTCGAAGCAACTGCACC	236	NM_071106.8	[3]
DAF-18	*daf-18*	ACATGCTGCGTCAGGATTAT	AAGGAATTGTCCGATGGTACTT	145	NM_067525.6	Designed by the research team

* All the sequences have been obtained from https://www.wormbase.org and have been validated with their access number using the NIH databases https://blast.ncbi.nlm.nih.gov/blast.cgi

1. Surco-Laos, F.; Cabello, J.; Gómez-Orte, E.; González-Manzano, S.; González-Paramás, A.M.; Santos-Buelga, C.; Dueñas, M. Effects of O-methylated metabolites of quercetin on oxidative stress, thermotolerance, lifespan and bioavailability on *Caenorhabditis elegans*. Food Funct. 2011, 2, 445-56. doi: 10.1039/c1fo10049a
2. Martinez-Finley E.J.; Caito S.; Slaughter J.C.; Aschner, M. The Role of *skn-1* in methylmercury-induced latent dopaminergic neurodegeneration. Neurochem Res. 2013,38, 2650-60. doi: 10.1007/s11064-013-1183-0.
3. Zhang, J.; Lu, L.; Zhou, L. Oleanolic acid activates daf-16 to increase lifespan in *Caenorhabditis elegans.* Biochem Biophys Res Commun. 2015, 25, 468, 843-849. doi: 10.1016/j.bbrc.2015.11.042.
4. Kern, A.; Ackermann, B.; Clement, A.M.; Duerk, H.; Behl, C. HSF1-controlled and age-associated chaperone capacity in neurons and muscle cells of *C. elegans*., PLoS One. 2010, 5, e8568. doi: 10.1371/journal.pone.0008568.
